# First Record of South American Sea Lion Predation on Non-Native Chinook Salmon at a Spawning Site in a Northern Patagonian River

**DOI:** 10.3390/biology15141147

**Published:** 2026-07-14

**Authors:** Cristóbal Garcés, Carlos Vega, Pablo Fierro

**Affiliations:** 1 Graduate School, Faculty of Sciences, Austral University of Chile, Valdivia 5110566, Chile; cgarces@alumnos.uach.cl; 2 Institute of Marine and Limnological Sciences, Faculty of Sciences, Austral University of Chile, Valdivia 5110566, Chile; cvega.vega11@gmail.com; 3 Centro Nacional de Investigación en Ríos, Invasiones y Sistemas (IRIS), Concepción 4030000, Chile

**Keywords:** biological invasions, freshwater ecosystems, predation

## Abstract

Chinook salmon *Oncorhynchus tshawytscha* (Walbaum, 1792) is a non-native species in southern South America, where self-sustaining populations are established. After spawning migrations, adults die and provide substantial subsidies to freshwater food webs. We report the first direct evidence of predation on spawning Chinook salmon by South American sea lions *Otaria byronia* (de Blainville, 1820) in a river of southern Chile, up to 160 km upstream from estuaries. Sea lions appear to be exploiting a novel prey resource, indicating behavioral plasticity with potential ecological consequences for native species and riverine trophic dynamics. We discuss the implications of this previously unreported predator–prey interaction for the ecology of invaded freshwater systems in South America.

## Simple Summary

Chinook salmon (*Oncorhynchus tshawytscha*) is an invasive fish that is now widespread in southern South America. Its establishment has created new ecological interactions in both freshwater and marine environments. Here, we report the first documented observation of a South American sea lion (*Otaria byronia*) preying on spawning Chinook salmon in a freshwater river of southern Chile, approximately 160 km from the sea and well beyond the species’ typical coastal habitat. This observation coincides with numerous records of sea lions occurring in inland rivers and lakes across southern South America over the last decade, overlapping the current distribution of Chinook salmon. Our findings highlight a previously unrecognized predator–prey interaction and provide new insights into the ecological consequences of biological invasions in South American freshwater ecosystems. These observations highlight the need for continued monitoring of invasive salmon impacts in South American freshwater ecosystems.

## 1. Introduction

Anadromous salmon species were intentionally introduced into the Southern Hemisphere multiple times beginning in the second half of the 19th century to establish commercial fisheries [1]. In South America, early introductions of Chinook salmon *Oncorhynchus tshawytscha* (Walbaum, 1792), based on eggs and juveniles imported from the United States during the 1920s and 1970s, appeared to be unsuccessful, as there was no evidence of natural reproduction in the stocked river systems [2,3]. However, experimental ocean-ranching programs in southern Chile during the late 1970s and early 1980s led to the first natural returns of Chinook salmon from the Pacific Ocean to Chilean rivers for spawning between 1979 and 1991 [4,5]. Subsequent escapes from salmon aquaculture cages in the 1990s accelerated the species’ spread [6], allowing Chinook salmon to establish reproducing populations throughout Patagonia. Within just 25 years, the species has rapidly expanded, establishing self-sustaining populations throughout most watersheds of southern Chile (39–53° S) and southern Argentina [5,7], becoming one of the most successful freshwater fish invasions in the Southern Hemisphere.

The establishment of Chinook salmon has triggered novel trophic interactions across terrestrial, freshwater, and marine ecosystems [8]. In freshwater environments, negative impacts on native fish assemblages have been reported, mainly through predation and competition [9]. In marine ecosystems, trophic overlap through shared prey with native predators such as the Magellanic penguin *Spheniscus magellanicus* (Forster, 1781) has also been documented [10]. More recently, studies have highlighted the role of spawning migrations in transferring marine-derived nutrients into riverine food webs through the deposition and decomposition of post-spawning carcasses, gametes, and excretory products, which subsidize aquatic and riparian consumers [8,11,12,13]. However, before becoming nutrient subsidies, spawning migrations also represent a seasonal pulse of live, energy-rich biomass that may provide novel foraging opportunities for native top predators capable of exploiting adult salmon.

**Figure 1:**
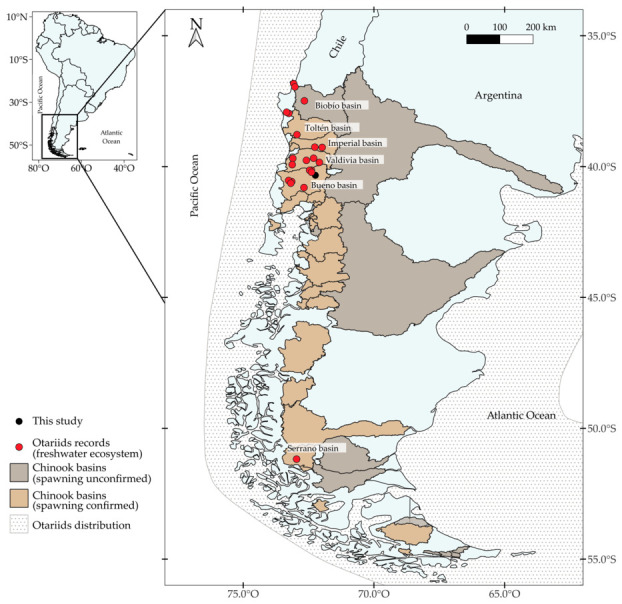
Otariid records in river basins of southern South America. Basins overlapping with the distribution of Chinook salmon (*Oncorhynchus tshawytscha*) are highlighted in grey and brown. All records correspond to *Otaria byronia*, except for one in the Serrano River basin in 2025, which involved an individual of *Arctocephalus australis*. Otariid records were obtained from press media and social networks (see Table S1). Chinook salmon distribution based on Figueroa-Muñoz et al. (2023) [7].

Here, we report the first record of Chinook salmon (*O. tshawytscha*) predation by South American sea lions *Otaria byronia* (de Blainville, 1820) observed in inland freshwaters, up to 160 km from the coast. We associate this behavior with the recent increase in reports of sea lions occurring in rivers and lakes over the past decade, overlapping the current distribution of Chinook salmon (Figure 1; see Supplementary Content, Table S1 for full data). We also discuss the potential ecological implications of this novel trophic interaction.

## 2. Materials and Methods

### 2.1. Review of Freshwater Records of Sea Lions

Records of South American sea lions (*O. byronia*) in inland freshwater systems were compiled through a targeted search of publicly available sources, including scientific literature, news media, and social media platforms (e.g., Facebook, X/Twitter, and Instagram). Searches were conducted using combinations of Spanish keywords (e.g., “sea lion”, “león marino” “lobo marino” “river”, “río” “freshwater”, “agua dulce” “lake”, “lago”, Chile, Argentina).

Only records providing sufficient spatial and temporal information (e.g., date and location) were included. Estuarine records and observations occurring within 10 km of the river mouth, measured along the river channel, were excluded. This threshold was selected to exclude estuarine mixing zones and ensure that only freshwater records were included, based on the reported extent of estuarine influence in the Valdivia and Toltén river estuaries [14,15]. Media reports were only considered when supported by verifiable visual evidence (photographs or videos). All records were georeferenced and used to generate the distribution map shown in Figure 1 and Table S1.

### 2.2. Field Monitoring in the Riñinahue River

Between 2023 and 2026, we conducted annual monitoring of Chinook salmon spawning in the Riñinahue River (40°19′57″ S, 72°14′22″ W), southern Chile (Figure 1). Monitoring consisted of bimonthly field surveys, each lasting 1–2 consecutive days, with approximately 4–8 h of fieldwork per day. The primary objective of these surveys was to sample benthic macroinvertebrates, fish assemblages, and water quality in spawning and reference reaches, while observations of Chinook salmon and associated ecological interactions, including the presence of potential predators and scavengers, were recorded opportunistically. The study reach was surveyed using a combination of drone flights, walking surveys along the riverbanks, and kayak surveys, allowing comprehensive coverage of the monitored section.

**Figure 2:**
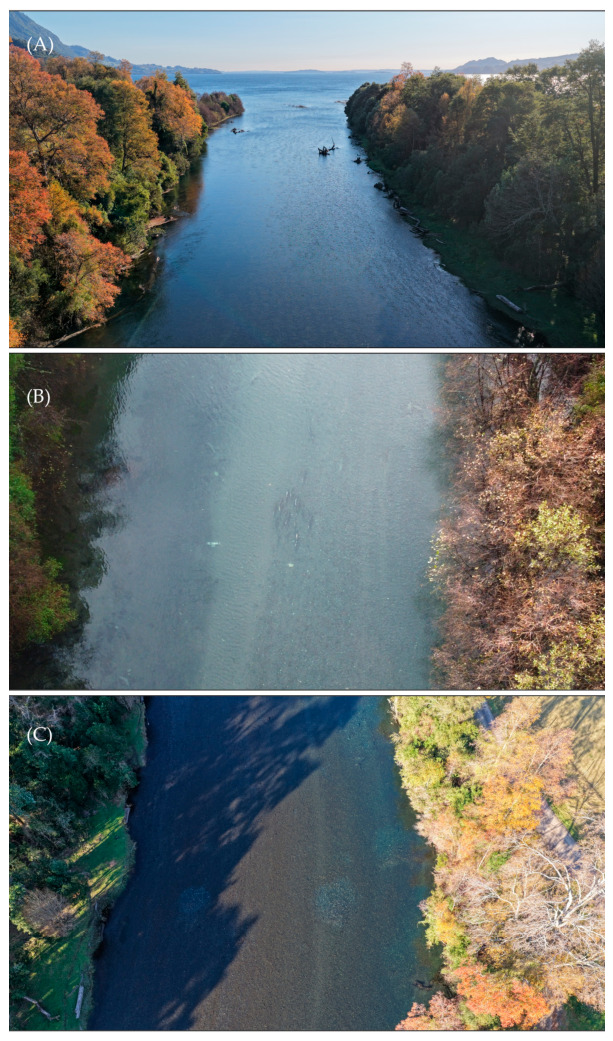
Study site in the Riñinahue River, Bueno River Basin. (**A**) Panoramic view of the study site, where the Riñinahue River flows into Lake Ranco. (**B**) School of spawning Chinook salmon (*Oncorhynchus tshawytscha*) in the Riñinahue River. (**C**) Salmon redds (nests) are visible on the riverbed.

The study reach is located approximately 164 km upstream from the Pacific Ocean. The monitored river section extends for approximately 1250 m, measured from its confluence with Ranco Lake to the downstream section of Riñinahue falls, a natural barrier that prevents upstream migration of Chinook salmon. The river ranges from 0.2 to 2 m in depth, has an average width of approximately 50 m, and at an elevation of 76 m as.l. Water clarity remains high throughout the year, with dissolved oxygen concentrations consistently exceeding 11 mg L^−1^ (100% saturation). Discharge varies seasonally, ranging from 22 m^3^ s^−1^ during the low-flow period to 42 m^3^ s^−1^ during high flow conditions (unpublished data). The watershed is characterized by a temperate rainy climate, with annual precipitation of 1800–2500 mm and mean temperatures ranging from 9–18 °C [16]. In this river, Chinook salmon spawning typically occurs between April and early June, with timing influenced by the onset of autumn rainfall. During the spawning season, salmon construct spawning nests (redds) in the river and remain there until they die (Figure 2).

The presence and hunting behavior of *O. byronia* were documented using high-resolution digital photography and video recordings. Species identification was based on external morphological characteristics visible in photographs and videos. *Arctocephalus australis* (Zimmermann, 1783) was distinguished from *O. byronia* primarily by the shape of the head and muzzle. *A. australis* has a slender head with a distinctly elongated, pointed muzzle, whereas *O. byronia* has a broader, more robust head with a short, blunt muzzle [17]. When photographic evidence was insufficient for reliable species identification, records between 39° and 40° S were assigned to *O. byronia*, given the absence of confirmed records of *A. australis* within this geographic range [18].

## 3. Results

### 3.1. Freshwater Records of Sea Lions

We compiled 23 records of otariids in freshwater ecosystems between 2013 and 2026. All but one record corresponded to *O. byronia*; the exception was a single *Arctocephalus australis* recorded in the Serrano River basin (Figure 1). The single record of *A. australis* was supported by high-quality photographs and identified by specialists from SERNAPESCA and CEQUA, as reported by multiple news sources. Most occurrences were reported in basins where Chinook salmon are present, with the highest number of records in the Bueno River basin (*n* = 7) and the Valdivia River basin (*n* = 5) (Table S1).

### 3.2. Field Observations in the Riñinahue River

**Figure 3:**
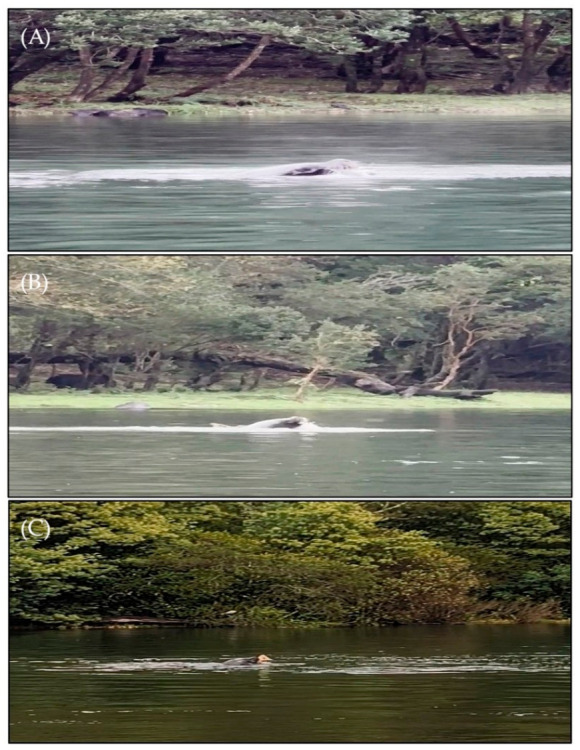
Video frames showing a South American sea lion (*Otaria byronia*) preying on spawning Chinook salmon (*Oncorhynchus tshawytscha*) in the Riñinahue River, 164 km upstream from the Pacific Ocean (Lake Ranco basin, Chile). (**A**) Subadult male moving upstream before capturing prey (Video S1). (**B**) Individual capturing a Chinook salmon and surfacing with it (Video S2). (**C**) Individual consuming a Chinook salmon, caudal fin visible (Video S3).

We recorded *O. byronia* in the Riñinahue River in two consecutive years during the Chinook salmon spawning season (Supplementary Videos S1–S5). In 2025, sea lion was observed in March and May, and in 2026 in June. On 28 March 2025, 21 May 2025 and 22 May 2025, we observed a subadult male *O. byronia* actively foraging in the river during daylight hours (10:00–18:00) (Figure 3; Supplementary Videos S1–S3). On 21 May 2025, we documented the predation of at least two adult Chinook salmon within a 1 km river section. The sea lion pursued salmon in shallow reaches (1–2 m depth), capturing live adult individuals at mid-body, and exhibited a prey-handling behavior, repeatedly striking the fish against the water surface before consumption, presumably to facilitate ingestion (Supplementary Video S3).

## 4. Discussion

Our observations provide the first evidence of *O. byronia* consuming Chinook salmon in freshwater habitats of South America. This interaction is likely facilitated by the high seasonal biomass of salmon during spawning migrations, representing an energetically valuable prey resource. Comparable behaviors have been documented in the Columbia River, USA, where pinnipeds prey heavily on salmonids at Bonneville Dam (~235 km upstream), substantially increasing salmon mortality [19]. Predation on Atlantic salmon by grey seals has also been reported in the Penobscot River, ME, USA [20], and additional evidence from the Columbia River indicates pinniped-inflicted injuries on migrating Chinook and sockeye salmon runs [21].

Recent studies have highlighted the role of spawning Chinook salmon as vectors of marine-derived nutrient subsidies to freshwater ecosystems. For instance, consumption of salmon eggs by other invasive fishes such as rainbow trout *Oncorhynchus mykiss* (Walbaum, 1792) has been reported in Chilean rivers [13]. Likewise, carcasses of spawned-out salmon serve as food for birds, mammals, and terrestrial invertebrates along riverbanks [8]. However, predation on live Chinook salmon during freshwater spawning migrations has not previously been reported in South America.

While predation on introduced salmon by sea lions has been documented in marine environments [22], our observations reveal a behavioral and spatial shift in foraging activity, with *O. byronia* exploiting Chinook salmon in freshwater habitats up to 160 km inland. This finding represents a novel freshwater foraging behavior and an opportunistic expansion of the species foraging range in response to an invasive prey, generating new predator–prey interaction with potentially important consequences for ecosystem structure and function [23].

Pinnipeds are known to develop individual foraging specializations that can spread socially, potentially leading to the expansion of this behavior among conspecifics [24,25]. A similar process was reported in southern Chile, where predation by sea lions on black-necked swans *Cygnus melanocoryphus* (Molina, 1782) in the Cruces River wetland rapidly escalated from a single individual in 2018 to mass mortality events (up to 133 individuals preyed upon in a single month) [25].

Although *O. byronia* may contribute to the regulation of invasive salmonids [22], rising predation pressure could indirectly affect native species through apparent competition [26]. During inland forays, sea lions may also feed on small native fishes, e.g., *Odontesthes* spp., *Basilichthys microlepidotus* (Jenyns, 1842), other introduced salmonids (*O. mykiss*, *Salmo trutta* Linnaeus, 1758), and waterbirds *Chroicocephalus maculipennis* (M.H.K. Lichtenstein, 1823) and *Phalacrocorax brasilianus* (J.F. Gmelin, 1789). Furthermore, inland incursions may facilitate the transport of parasites and pathogens into freshwater ecosystems. Pinnipeds harbor several parasite genera with zoonotic potential (e.g., *Entamoeba*, *Balantidium*, *Diphyllobothrium*, and *Anisakis*) as well as zoonotic bacteria (e.g., *Clostridium*, *Escherichia*, *Vibrio*, *Yersinia*, and *Salmonella*) [27,28]. Recent studies also suggest that pinnipeds may act as reservoirs and dispersal hosts of highly pathogenic avian influenza virus (H5N1), highlighting their potential role in pathogen transmission among wildlife [29].

In South America, Chinook salmon support a growing recreational fishery in freshwater ecosystems in Chile and Argentina, where fishing is prohibited during the spawning period (approximately May to November). Commercial fisheries are currently limited to small-scale artisanal operations in the Toltén and Valdivia river estuaries (Chile), although illegal and subsistence fishing also occurs during the spawning migration [30]. Notably, Chinook salmon have few significant natural predators capable of targeting live adults during their upstream migration. In this context, sea lion incursions and predation may represent an emerging ecological interaction with potential implications for fishery management and riverine ecosystem dynamics.

Given the rapid expansion of Chinook salmon and the ecological plasticity of *O. byronia*, long-term monitoring of these emerging predator–prey interactions is essential. Understanding how invasive salmon subsidies influence top predator behavior will provide valuable insights into the ecological consequences of biological invasions in South American freshwater systems.

## 5. Conclusions

We report the first confirmed observation of South American sea lions (*Otaria byronia*) preying on spawning Chinook salmon (*Oncorhynchus tshawytscha*) in a freshwater river in southern Chile. This record extends the known inland foraging range of *O. byronia* and documents a novel predator–prey interaction within invaded freshwater ecosystems.

These observations, together with reports of sea lions in inland waters, suggest emerging ecological interactions associated with the spread of Chinook salmon. Continued monitoring is needed to evaluate the frequency and ecological consequences of these interactions.

## Data Availability

All data supporting the findings of this study are available in Table S1 and Supplementary Videos, and have been deposited in Figshare at https://doi.org/10.6084/m9.figshare.3026721.

## Supplementary Materials

The following supporting information can be downloaded at: https://doi.org/10.6084/m9.figshare.30267214 (Online resource posted 8 July 2026). Table S1. Records of otariids in inland waters (lakes, rivers, and wetlands) of Chile compiled from scientific literature, press media, and social media sources (X/Twitter, Facebook, Instagram). Dataset available as XLSX file. Video S1. Subadult male South American sea lion (*Otaria byronia*) swimming in the Riñinahue River, southern Chile (21 May 2025; 40°19′57″ S, 72°14′22″ W). MP4 file (1920 × 1080). This video is part of a series documenting inland occurrence of sea lions up to 164 km upstream in the Riñinahue River. Video S2. Subadult male South American sea lion (*Otaria byronia*) holding a Chinook salmon (*Oncorhynchus tshawytscha)* between its jaws in the Riñinahue River, southern Chile (21 May 2025; 40°19′57″ S, 72°14′22″ W). MP4 file (1920 × 1080). This video documents predation behavior in freshwater habitat. Video S3. Subadult male South American sea lion (*Otaria byronia*) consuming a Chinook salmon (*Oncorhynchus tshawytscha*) in the Riñinahue River, southern Chile (21 May 2025; 40°19′57″ S, 72°14′22″ W). The individual shakes and ingests the posterior portion of the fish, with the caudal fin visible. MP4 file (1920 × 1080). Video S4. Subadult male South American sea lion (*Otaria byronia*) swimming in the Riñinahue River, southern Chile (28 March 2025; 40°19′57″ S, 72°14′22″ W). MP4 file (1920 × 1080). Video S5. Subadult male South American sea lion (*Otaria byronia*) swimming in the Riñinahue River, southern Chile (15 June 2026; 40°19′57″ S, 72°14′22″ W). MOV file (1920 × 1080).
